# GlycoMaple: recent updates and applications in visualization and analysis of glycosylation pathways

**DOI:** 10.1007/s00216-024-05594-1

**Published:** 2024-10-17

**Authors:** Wei-Ze Kong, Morihisa Fujita

**Affiliations:** Institute for Glyco-core Research (iGCORE), Gifu University, Gifu, 501-1193 Japan

**Keywords:** Glycosylation, GlycoMaple, Glycogene, Glycan biosynthesis

## Abstract

Post-translational modifications including glycosylation, phosphorylation, and lipidation expand the functionality and diversity of proteins. Protein glycosylation is one of the most abundant post-translational modifications in mammalian cells. The glycosylation process is regulated at multiple steps, including transcription, translation, protein folding, intracellular transport, and localization, and activity of glycosyltransferases and glycoside hydrolases. The glycosylation process is also affected by the concentration of sugar nucleotides in the lumen of the Golgi apparatus. Unlike the synthesis of other biological macromolecules, such as nucleic acids and proteins, glycan biosynthesis is not a template-driven process. In addition, the chemical complexity of glycan structures makes the glycosylation network extraordinarily intricate. We previously developed a web-based tool specially focused on glycan metabolic pathways known as GlycoMaple, which is able to easily visualize and estimate glycosylation pathways based on gene expression data. We recently updated GlycoMaple to incorporate the new genes and glycosylation pathways. Here, we introduce and discuss the uses and upgrades of GlycoMaple.

## Introduction

Glycosylation is catalyzed by a series of glycosyltransferases (GTs) and glycoside hydrolases (GHs) in sequential steps. The polysaccharides are covalently linked to proteins or lipids to expand their functionality and diversity [1]. Glycosylation alters the physical and chemical properties of proteins and lipids, which affects their solubility, stability, and interactions with other molecules. Glycans, a class of macromolecules documented in various metabolic pathway databases like the KEGG database [2], have important roles in multiple biological processes, including cell adhesion and cell-cell interaction [3]. Determining glycan structures in cells or tissues of interest is valuable for understanding physiological functions and aiding in disease diagnosis. In particular, differences in glycan structures between normal and cancerous tissues as well as changes in glycan structures under different culture conditions, will contribute to the development of biomarkers for disease and differentiation [4–6]. Nonetheless, it is challenging to obtain a systematic overview of the numerous glycosylation pathways because of the intricate metabolic network, the similarity in chemical composition, and the non-template-driven nature of glycan synthesis [3]. Currently, the glycan structures of cells or tissues are determined primarily by mass spectrometry (MS) and high-performance liquid chromatography, which require skilled personnel and advanced analytical methods. The analysis of glycans with charges or modifications and high molecular weight glycan structures is particularly challenging. As an alternative method, it may be possible to use informatics to visualize glycan metabolic pathways and estimate glycan structures.

GlycoMaple is a web-based human glycosylation mapping tool that can visualize more than 20 different glycosylation pathways based on gene expression profiles in cells or tissues [7]. It has been integrated into the GlyCosmos Portal [8] making it accessible through this platform (https://glycosmos.org/glycomaple/index). The primary goal for developing GlycoMaple was to create a user-friendly visualization and estimation tool for glycosylation pathways that could be easily understood by researchers from various backgrounds. GlycoMaple enables researchers to gain comprehensive insight into glycan structures and their biosynthetic processes/pathways, ultimately advancing our understanding of the glycosylation process. In this Trends Article, we provide an overview and discuss the GlycoMaple tool as well as describe the emerging in silico models for glycan-related pathways.

## Overview of GlycoMaple

**Fig. 1:**
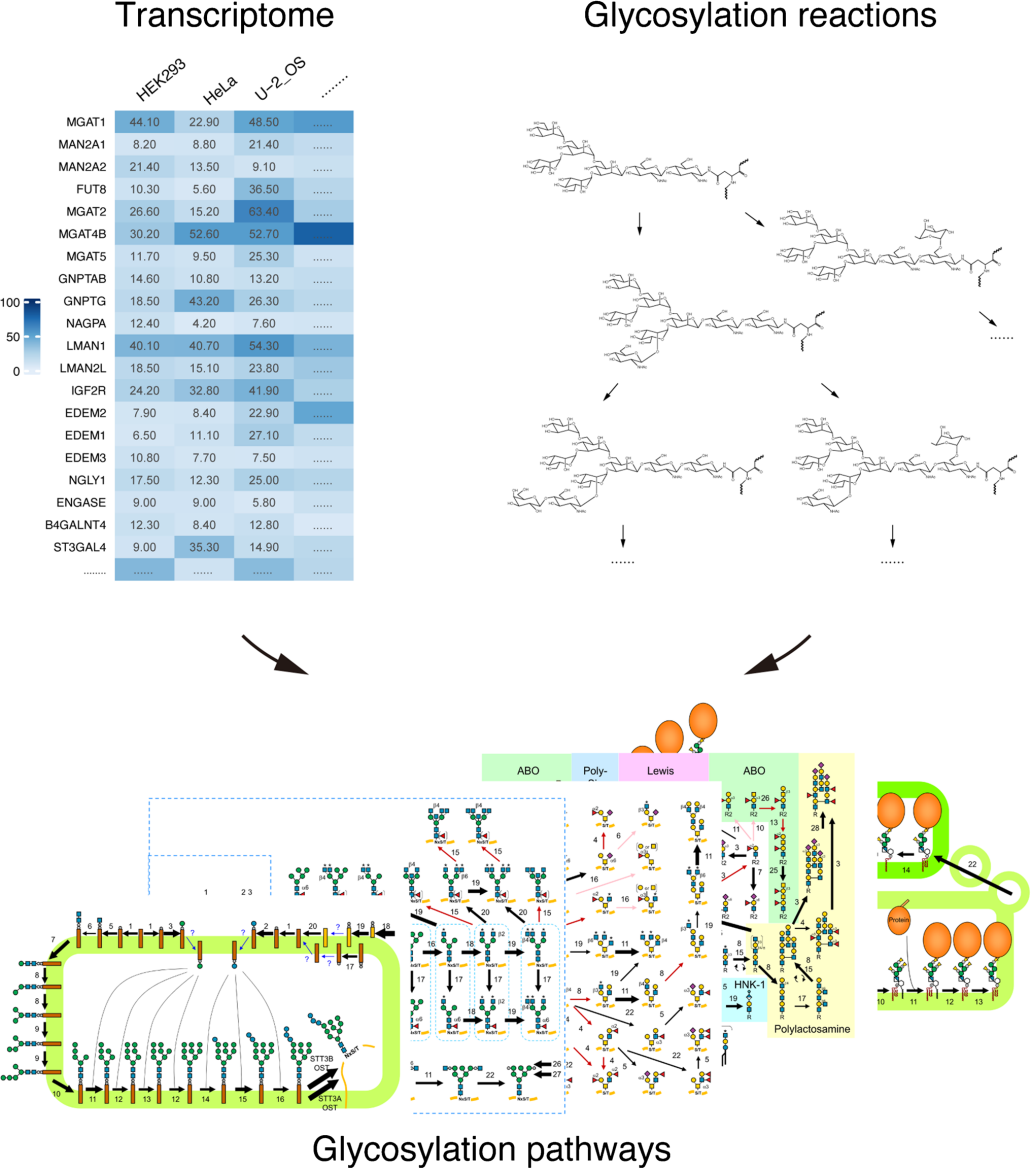
Overview of the GlycoMaple tool. GlycoMaple is a tool to visualize glycosylation pathways and estimate glycan structures based on gene expression. The tool integrates genes required for each reaction in glycan metabolic pathways (upper right). Once users obtained gene expression data from cells/tissues of interest using RNA-seq (upper left), GlycoMaple visualizes over 20 kinds of glycan metabolic pathways (bottom) in humans by uploading the gene expression data. All glycan structures are represented by the Symbol Nomenclature for Glycans (SNFG)

**Fig. 2:**
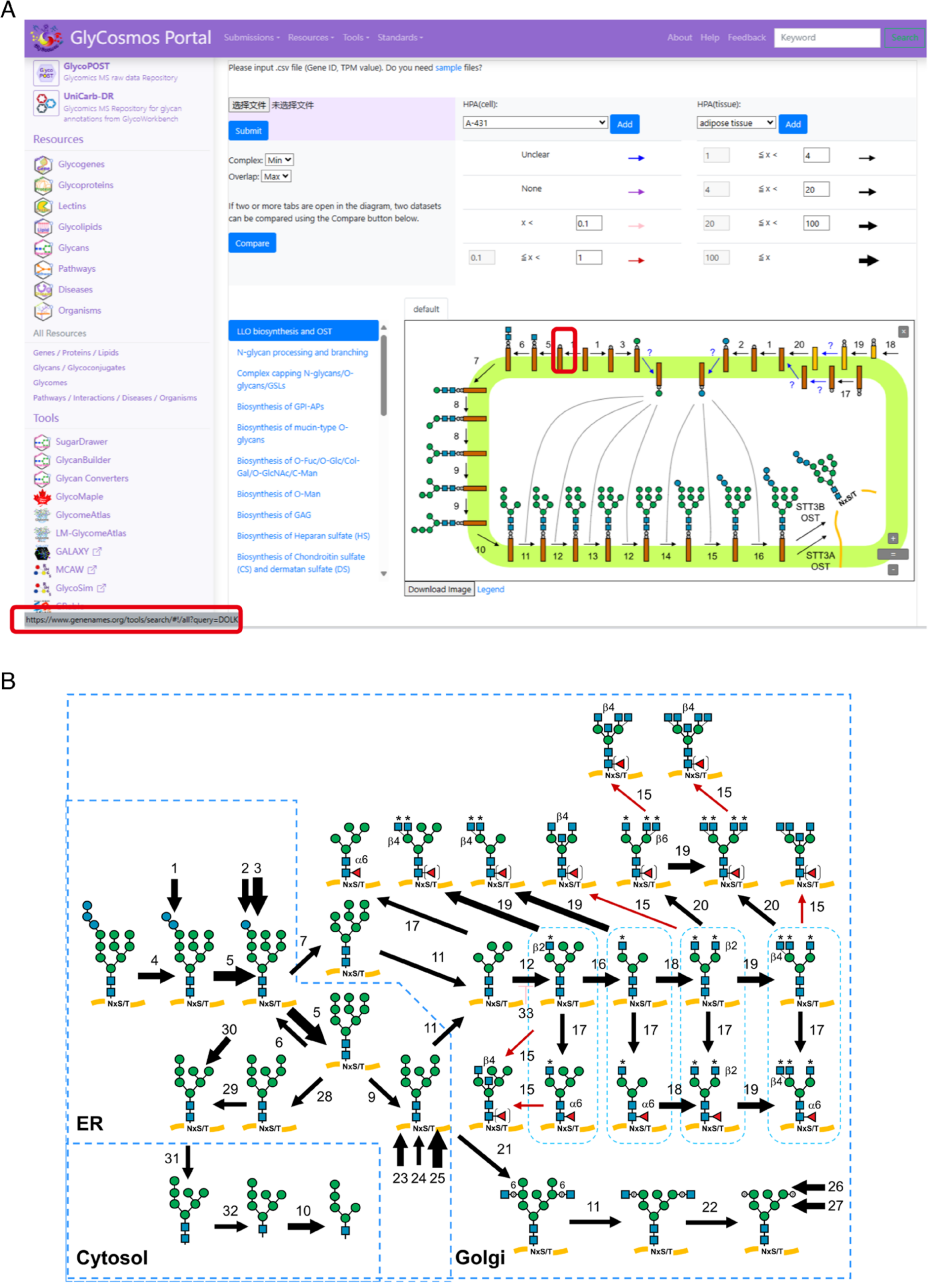
Use of the GlycoMaple tool. **A** An illustration of the interactive features on the GlycoMaple website. The red squares in an arrow and at the bottom left indicate the gene name responsible for the reaction, which are linked to the HGNC database website. **B** Visualization of the N-glycan processing pathway in HEK293 cells based on the gene expression profile. When users upload their own gene expression data or select data from cells or tissues in the Human Protein Atlas (HPA) database, the arrows for each reaction are updated based on the gene expression profiles. The thickness of the black arrows represents the expression levels of the genes required for the reactions. The red and pink arrows indicate that the expression of the genes involved in those reactions is either limited or absent, respectively.

GlycoMaple enables a clearer understanding of glycosylation pathways and supports the robust estimation of glycosylation dynamics when analyzing multiple pathways in different cells. This is particularly useful for researchers aiming to elucidate glycosylation pathways from the perspective of gene expression to enhance the precision and efficiency of glycosylation-related studies. By 2024, GlycoMaple included 21 glycan metabolic pathways and over 1000 glycan-related genes, which cover nearly all known glycosylation processes in human-derived cells (Figure 1). To facilitate efficient communication and enhance our understanding, all glycan structures in GlycoMaple are represented by the Symbol Nomenclature for Glycans (SNFG) [9, 10]. In addition, an interactive feature enables users to see the names of the genes encoding the enzymes that catalyze specific reactions by hovering a mouse pointer over the corresponding arrows in the pathway plots (Figure 2A). Clicking on an arrow directs users to the Human Genome Organization (HUGO) Gene Nomenclature Committee (HGNC) database [11, 12], where they can access more detailed information about specific genes.

GlycoMaple facilitates the straightforward interpretation of RNA-seq data in the context of glycosylation pathways, enabling the connection of RNA-seq data to biologically relevant phenotypes. Because of the significant decrease in its cost over the past decade, RNA-seq has become one of the most important tools for gathering omics data in the database. It provides global insight into the biosynthesis processes based on gene expression profiles [13]. Although studies have shown that gene expression levels are not always correlated with the abundance of the corresponding protein [14–16], gene expression data could be used to estimate the presence or absence of specific glycan structures by analyzing the receiver operating characteristic (ROC) curve and calculating the area under the curve (AUC). In the mucin-type O-glycan biosynthesis pathway, the optimal TPM threshold was determined as this value yielded the best Youden’s *J* statistic, which determines the cutoff values for prediction, and F1 score that is the harmonic means of sensitivity and precision [7]. In the default setting, the threshold is set as transcript per million reads (TPM) = 1 for a gene required in the reaction step. For example, when inputting the RNA-seq data of HEK293 cells into GlycoMaple, the N-glycan processing pathway is displayed as shown in Figure 2B. The reactions with gene expression above the threshold are indicated by black arrows, and the thickness of the arrows corresponds to the intensity of the expression. The expression of MGAT3, which is involved in the reaction at Step 15 (formation of bisecting GlcNAc), is below the threshold and is indicated by a red arrow, suggesting that few bisecting N-glycan structures exist in HEK293 cells. When representing gene expression in each reaction as an arrow, it is difficult to display the isoenzymes as well as the enzyme complexes that are involved in one reaction in the glycosylation process. GlycoMaple uses the maximum gene expression to show a reaction involving multiple isoenzymes by default, whereas the minimum expression value among the subunit genes is used for genes encoding enzyme complexes. It also offers alternative options, such as using the average, minimum or maximum value for the isoenzymes and subunits of complex enzymes (Figure 2A).

**Fig. 3:**
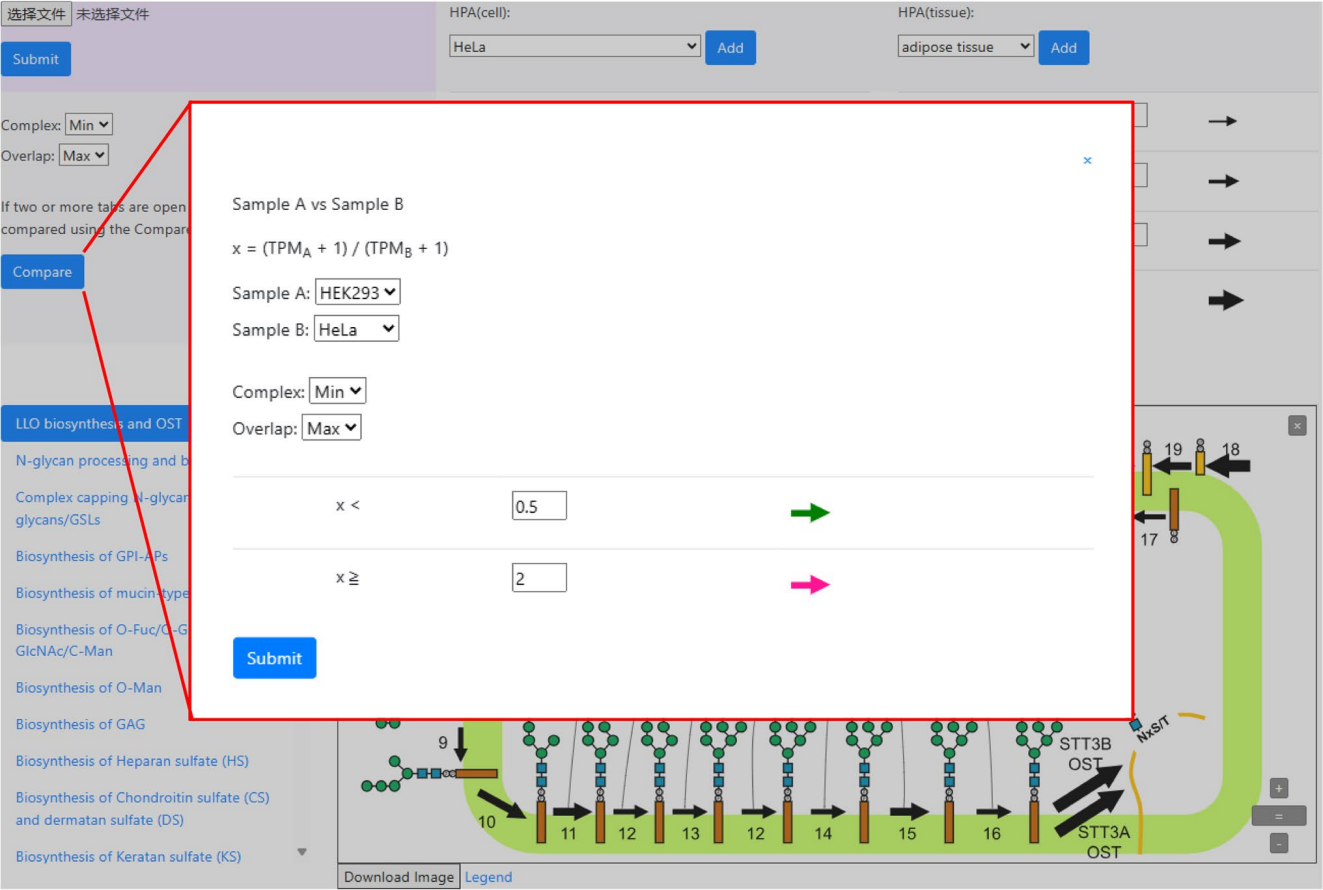
A comparison function in GlycoMaple. By clicking the “Compare” button, the user can choose two gene expression data of interest, which are compared. After setting the threshold, the “Submit” button is clicked to start the comparison

RNA-seq data is often used to identify differential gene expression for gaining biological insights into pathways and processes. To provide more insight into the various glycosylation pathways, GlycoMaple possesses a comparison function (Figure 3). After conducting a comparison, genes from different samples that exhibit fold changes exceeding the setting threshold are highlighted in pink or green, indicating higher or lower expression, respectively. This feature allows for an intuitive and nuanced understanding of gene expression changes across samples.

## Update on human GlycoMaple

Since GlycoMaple was released in 2021, extensive studies have been conducted that have resulted in new insights regarding the glycosylation pathway, which has necessitated updates to GlycoMaple. The following updates have been made based on the recent findings.

**Fig. 4:**
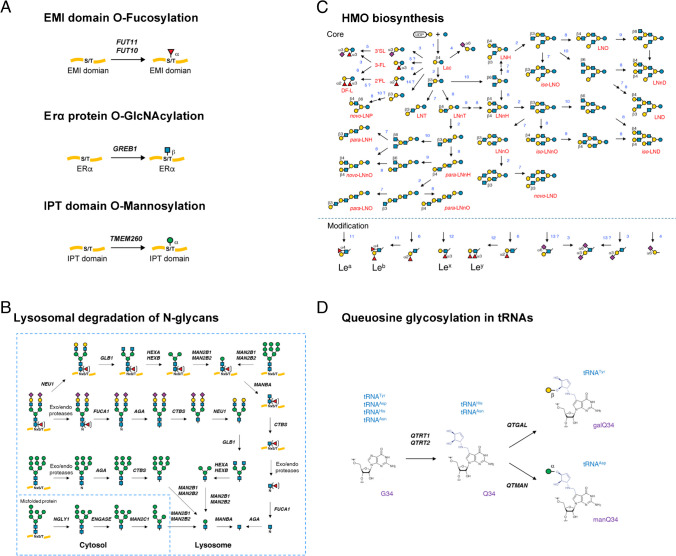
GlycoMaple updates. **A** New reactions added to GlycoMaple, including EMI domain-specific O-fucosylation reaction, ERα protein O-GlcNAcylation, and IPT domain-specific O-mannose modification. **B** Updated lysosomal degradation pathway. **C** HMOs biosynthetic pathway. **D** Queuosine tRNAs glycosylation pathway

In the context of the O-fucose biosynthetic pathway, a new EMI domain-specific O-fucosylation reaction catalyzed by FUT10 and FUT11 has been added [17]. For O-GlcNAcylation, apart from OGT, GREB1 is another gene that has been identified that encodes O-GlcNAc glycosyltransferase acting on the ERα protein in mammalian cells [18]. Another novel O-mannosylation, which occurs on IPT domains in plexins and hepatocyte growth factor receptors, is catalyzed by TMEM260 [19]. These reactions have now been included in GlycoMaple (Figure 4A).

Regarding the lysosomal N-glycan degradation pathway, the protein parts were initially depicted as being digested by exo/endo proteases in the previous version of GlycoMaple. Subsequently, the glycans were further processed by various glycosidases until complete degradation occurred; however, recent reports suggest that degradation can also occur while N-glycans are covalently linked to proteins [20]. For example, paucimannosidic proteins, which are generated in lysosomes, exist in mammalian cells. This finding prompted us to significantly update the lysosomal degradation pathway plot (Figure 4B).

Human milk oligosaccharides (HMOs) are a family of structurally diverse unconjugated glycans that are highly abundant and unique to human milk. As the third most abundant component of human milk after lactose and lipids, HMOs represent the first prebiotics that humans are exposed to, which play an important role in shaping the infant gut microbiome [21]. Moreover, in the gut, many pathogens first attach to the glycocalyx of epithelial cells when invading host tissues. HMOs may act as “decoys” for many of these pathogens because of their similar composition [22–24]. The updated GlycoMaple now includes the HMO biosynthetic pathway (Figure 4C). In total, 39 core structures and 6 terminal modifications are illustrated in the pathway plot. Thirty genes encoding the biosynthetic enzymes are involved in the HMO biosynthetic pathway. Because of insufficient direct evidence proving their involvement in HMO biosynthesis, the enzyme specificity for some genes is inferred from other known glycan biosynthetic pathways. The unknown genes involved in HMO biosynthetic pathway are indicated with question marks next to the arrows.

Recently, QTGAL and QTMAN were identified as the genes encoding the enzymes that catalyze the queuosine galactosylation and queuosine mannosylation of tRNAs, respectively [25]. This discovery has revealed a new pathway known as “Queuosine glycosylation in tRNAs” into GlycoMaple (Figure 4D), which ensures the comprehensive coverage of all known glycosylation reactions. These updates enhance the accuracy and comprehensive nature of GlycoMaple and reflect the latest advancements in glycosylation research.

## Glycosylation pathway estimation using GlycoMaple

**Fig. 5:**
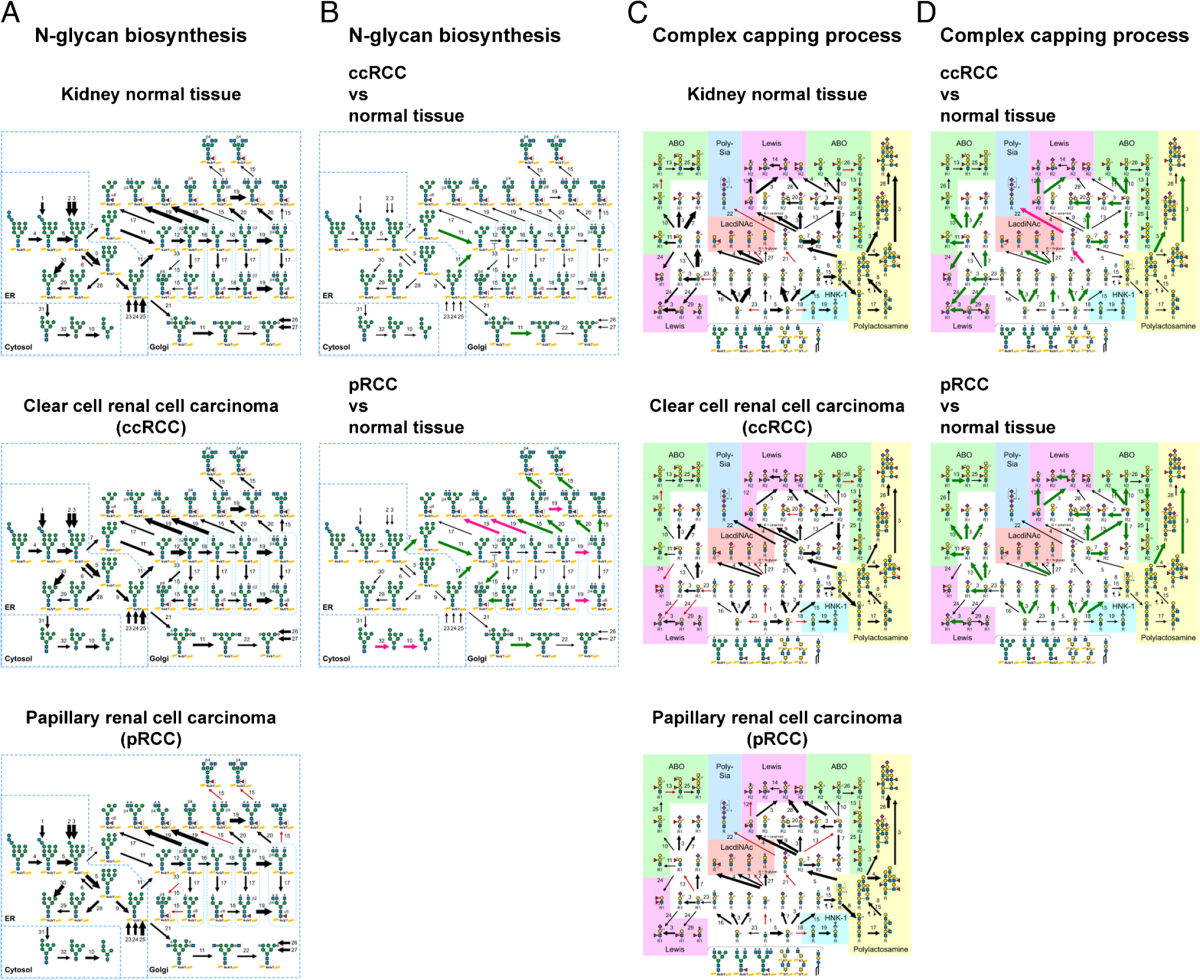
GlycoMaple analysis of normal kidney tissue, ccRCC, and pRCC in TCGA. **A**, **C** N-glycan processing (**A**) and complex capping structure (**C**) pathways in kidney normal tissue, clear cell renal cell carcinoma (ccRCC), and papillary renal cell carcinoma (pRCC) are visualized, respectively. The thickness of the black arrows represents the expression levels of the genes required for the reactions. Red and pink arrows indicate that the expression of the genes involved in those reactions is either limited or absent, respectively. **B**, **D** Comparison of the N-glycan processing (**B**) and complex capping structure (**D**) pathways in ccRCC or pRCC versus kidney normal tissue are shown, respectively. The dark pink and green arrows represent reactions where the expression of the genes involved has increased to more than double or decreased to less than half, respectively, in cancer tissues compared to normal tissues

Using updated GlycoMaple, RNA-seq data from TCGA database were analyzed. We selected gene expression data from clear cell renal cell carcinoma (ccRCC), papillary renal cell carcinoma (pRCC), and normal kidney tissue, which were processed by Xena Hub [32]. The median values of the gene expression data in different samples were uploaded into GlycoMaple and visualized in different glycosylation pathways (Figure 5A and C). GlycoMaple enables a comparison of the gene expression profiles between ccRCC, pRCC, and normal tissue (Figure 5B and D).

In the N-glycan biosynthetic pathway, a low expression of the *MGAT3* gene, which is responsible for the biosynthesis of the bisecting structure in N-glycan, was observed in the pRCC, but not in the ccRCC (Figure 5A). This suggests that a bisecting structure is rare in pRCC. In addition, *MAN1A1*, *MAN1A2*, and *MAN1C1* expression was significantly decreased in both ccRCC and pRCC compared with normal kidney tissue (Figure 5B). In a comparison between pRCC and normal kidney tissue, *MGAT4A* and *MGAT4B*, which are responsible for the biosynthesis of the β1,4-GlcNAc branch, were increased in pRCC, whereas the *MGAT5* and *MGAT5B* genes responsible for the biosynthesis of the β1,6-GlcNAc branch were decreased (Figure 5B). Examination of the complex capping structure revealed the expression of the *ST8SIA4* gene, which is required for the biosynthesis of the poly-sialic acid structure, was significantly upregulated in ccRCC, whereas its expression was limited in pRCC and normal kidney tissue (Figure 5C and D). Overall, these findings provide insight into the glycosylation pathways in ccRCC, pRCC, and normal kidney tissue, highlighting the potential differences in N-glycan branching and sialylation modifications.

## Disadvantage of GlycoMaple tool and further perspective

Although GlycoMaple provides valuable insight for the visualization and comparison of glycosylation pathways, certain limitations persist. Primarily, as a visualization tool, it is restricted to human-derived cell glycosylation pathways, limiting its applicability to other species commonly used in biological research, such as mice. Additionally, this tool offers qualitative rather than quantitative descriptions of the glycosylation pathways due to the decoupled relationship between gene expression, protein abundance, and glycan abundance data [14–16, 26]. Moreover, the changes in reactions are shown in the fixed pathways, confusing the interpretation in some cases particularly in glycan-related gene knockout cells, where glycosylation pathways could be different from the parental cells.

Future developments of GlycoMaple aim to expand its visualization and comparison capabilities to other species, such as mice, by integrating glycosylation pathways from various organisms, thereby enhancing its comprehensiveness. Additionally, further expansion of GlycoMaple seeks to provide quantitative descriptions of glycosylation pathways based on gene expression profile, with an initial emphasis on the N-glycosylation pathway. To achieve this, one example would be an absorbing Markov chain to model the N-glycosylation process. Absorbing Markov chain is a type of Markov chain model where certain states (absorbing states), once entered, cannot be left once entered. By omitting the protein to which N-glycans are attached and sugar nucleotides involved, glycosylation reactions and associated transport processes could be described as transitions from one glycan to another, with certain probabilities. The subsequent glycan structure depends solely on the current structure, independent of previous states, thus aligning with the Markov chain principle, where the future state is determined only by the present [27]. Consequently, the N-glycosylation pathway could be converted into a Markov transition matrix, enabling a quantitative description. This matrix will then be integrated with gene expression profile, allowing for quantitative estimation of N-glycan profile based on gene expression data. Additionally, the reconstruction function will be featured in subsequent development, allowing users to generate customized pathway plots tailored to specific knockout cell lines based on a defined set of enzymes, thereby avoiding confusion arising from fixed pathway representations.

## In silico models for glycosylation pathways

Over the past two decades, various in silico models have been developed to elucidate glycosylation pathways. The pioneering glycosylation model was based on kinetics, depicting the N-glycosylation pathway as a series of consecutive biochemical reactions with detailed kinetic parameters. It provides a quantitative description of each reaction based on biochemical reaction kinetics, calculating the abundance of each glycan by solving nonlinear equations. Several assumptions such as homogeneous Golgi compartments (cis, mid, trans, and TGN) and stable compartment component while cell growth have been incorporated into the model [28, 29]. Despite these simplifications and assumptions, a large number of parameters are still required.

Subsequently, the constraint-based reconstruction and analysis (COBRA) toolbox was provided for modeling the genome-scale metabolic networks, allowing for the analysis and prediction of phenotype outcomes [30, 31]. In the COBRA model, curated metabolic network is converted into the stoichiometric matrix (S) to provide a mathematical description [32]. Initially, under the steady-state assumption, the equation S · v = 0, where v represents the vector of reaction fluxes, defines a broad solution space. This space is then refined by incorporating various constraints, such as mass conservation, maximum enzyme capacity, and enzyme kinetics. While the refined solution space offers an unbiased description of potential reaction fluxes for a given organism under specific circumstances, optimization towards an objective function (e.g., maximum growth rate) determines a specific set of reaction fluxes [31]. Nevertheless, the intricacy of the glycosylation pathways, the challenge of obtaining constraint parameters, and the vast number of possible glycan structures make constructing the N-glycosylation network based solely on constraint-based methods exceedingly difficult. In 2016, a novel N-glycosylation model that integrated the Markov chain theory with constraint-based optimization methods was proposed. This model utilizes the stochastic process to capture the properties of N-glycosylation pathways and optimizes the parameters by converting the Markov-formed parameters into flux balance analysis (FBA) problem, demonstrating the capability to model the N-glycosylation profile on glycoproteins precisely [27]. Further development of this Markov chain–based model has shifted to alternative optimization methods rather than relying on the COBRA toolbox, thereby improving the prediction capabilities [33, 34]. Compared with kinetic-based models, Markov chain–based models significantly decrease the required parameters and necessitate less prior knowledge of glycosylation pathways, making them easier to apply to various cell types and even to other glycosylation pathways, such as the glycolipid biosynthesis pathway [35].

Furthermore, the machine learning model, in combination with the kinetic-based model, has been applied to predict protein glycosylation [36]. This approach harnesses the power of machine learning algorithms and broadens the scope of data formats applicable for model training, for example, gene expression data. Development of novel modeling approaches, such as Markov chain–based models and machine learning methods, holds great potential for advancing our understanding of glycosylation pathways and facilitating the control of glycan profile in plenty of pharmaceutical proteins.

## Outlook

Metabolic pathway databases, such as the KEGG database [2], play an important role in understanding, anticipating, and elucidating metabolic pathways in various organisms. While these databases offer comprehensive coverage of numerous organisms, they often lack detailed information in the glycosylation area because of the variability and intricate nature of glycosylation pathways. In this Trends Article, we summarize the development, application, and updates of GlycoMaple, a tool specially designed for visualizing glycan metabolic pathways to address these limitations. Despite its usefulness, GlycoMaple still has certain limitations, including issues with displaying glycans and their related pathways quantitatively. Future developments will focus on creating models capable of quantitatively describing relative glycan abundance based on gene expression data. There remain ample opportunities for further refinement and expansion of its capabilities. In conclusion, ongoing updates to GlycoMaple will ensure its alignment with the latest knowledge of glycosylation pathways.
